# Determinants of depression in primary caregivers of disabled older relatives: a path analysis

**DOI:** 10.1186/s12877-017-0667-1

**Published:** 2017-11-23

**Authors:** Rafael del -Pino-Casado, Pedro A. Palomino-Moral, Maria del Mar Pastor-Bravo, Antonio Frías-Osuna

**Affiliations:** 1 School of Health Sciences, Department of Nursing, University of Jaén, Jaén, Spain; 2 Lawrence S. Bloomberg Faculty of Nursing, University of Toronto, 155 College Street, Toronto, ON M5T 1P8 Canada

**Keywords:** Caregivers, Depression, Caregiving motives, Obligation

## Abstract

**Background:**

Despite the large literature analysing factors related to depression, several factors such as caregiving obligation and the interrelationships among the different variables relating to depression have been little studied. The current study aimed to analyse the effect of caregiving obligation (beliefs regarding obligation and social pressure) on depression, and the mediating effects of perceived burden on the relationship between stressors and depression, in primary caregivers of older relatives.

**Methods:**

Cross-sectional study design. A probabilistic sample of caregivers from Spain (*N* = 200) was used. The data collection was conducted in 2013 through structured interviews in the caregivers’ homes. The measures included sense of obligation for caregiving, perceived burden, stressors and depression.

**Results:**

Depression had a direct and positive association with perceived burden, behavioural problems, and social pressure, and it was indirectly related through perceived burden to behavioural problems, independence for the activities of daily living and beliefs of obligation.

**Conclusions:**

Our results support the multidimensional concept of obligation, suggesting the existence of both an external obligation (social pressure) and an internal obligation (beliefs of obligation); (b) our findings support the hypothesis that external obligation is related to negative caregiving consequences, while internal obligation protects from these consequences; and (c) our findings support the partial mediation of stressors on depression by perceived burden.

The relevance of the research to clinical practice includes the importance of understanding the perceived obligation of caregiving related to both internal and external sources of obligation.

## Background

In industrial countries, research on the family care of dependent elderly individuals is growing in importance, as evidenced by the increased numbers of aged, dependent individuals [1], the importance of informal care in the care of dependent elderly individuals, and the importance of family in informal care [2]. Both aging and dependence increase the demands involved in long-term care. The bulk of the care for elderly persons is family-focused, reflecting the broader truth that the system of care for dependency relies largely on the family.

Caring for a dependent relative has proven detrimental to the health of caregivers [3]. Among the health consequences for such caregivers, depression is prominent in both its magnitude and its significance. Several studies have examined the prevalence of depression in caregivers of elderly persons, placing its prevalence between 26 to 57% [4–6]. With the present study, we try to contribute to research supporting the prevention and early detection of depressive symptoms in the caregivers of dependent elderly.

### Relationship between stressor and depression

The occurrence of depression in caregivers is related to the stress of caregiving. Therefore, theoretical models used to explain the appearance of this depression are based on Lazarus and Folkman’s Transactional Stress Theory [7]. In this model, caregiver stress and its consequences are a result of the appraisal of the stress sources in the caregiving situation. Stress sources, or stressors, are defined by the needs of the care recipient: functional status, cognitive impairment and behavioural problems [8, 9]. The appraisal of the magnitude and implications of these stressors may lead to evaluations of perceived burden, and as a result, also to psychological outcomes such as depression among caregivers [10].

Perceived burden can be defined as a caregiver’s state, characterized by distress in several areas (caregiver’s health, psychological well-being, finances, social life and the relationship between caregiver and care-recipient), resulting from the caregiving situation [11]. Several studies have examined the relationship of previous factors with depression in the caregivers of dependent elderly, showing that stressors and perceived burden are positively correlated with depression [3, 12].

### Cultural factors and depression

Stress related models based on Lazarus and Folkman [7] have been criticized for not considering cultural factors of caregiving, despite the growing evidence showing the influence of these cultural aspects in the caregiving process [13]. These cultural factors can be defined as values, beliefs, and rules of a particular social group [8] and have been incorporated into theoretical models as determinants of caregiving consequences [13].

Specifically, the Mediterranean culture of care, in which this study was completed, is characterized by low female participation in the labour market, a high female participation in care, a positive attitude toward obligation to family care and a lower development of the formal care system. Caregivers have cultural motives for caregiving in general, and particularly obligation for caregiving plays an important role [8, 14].

### Mediating roles of perceived burden

Although obligation for caregiving has been moderately analysed in the caregiving literature [12, 13], little attention has been paid to the relationship between obligation and depressive symptoms [15]. Furthermore, previous studies suggest that the motives for caregiving [15] and in particular the obligation to provide care [8, 16] are multidimensional, including both an internal obligation (related to beliefs about the duty of care) and an external obligation (related to the social pressure to provide caregiving). Theoretically, the multidimensionality of obligation is consistent with the Ryan and Deci’s self-determination theory [17]. These authors classified motivation in two levels [17]: (a) internal motivation: identified regulation (the activity is judged to be valuable by the person) and integrated regulation (the identification with the activity was in harmony with other structures within the self), and (b) external motivation: external regulation (to satisfy an external demand) or “introjected regulation” (social pressure). Several studies [18] have shown that internal motivation tends to yield greater psychological health. In a similar way, results from Romero-Moreno et al. [15] suggest that internal motives may be related with less depressive symptoms. Thus, each dimension of obligation (internal obligation or beliefs of obligation and external obligation or social pressure) may relate differently to the consequences of care, with beliefs of obligation likely preventing depressive symptoms and social pressure likely causing depressive symptoms. Therefore, these issues also require further investigation.

Another little-studied issue is the interrelationships among the different variables relating to depressive symptoms. The variables related to informal care are often highly interrelated [3], but in most studies on informal care, researchers have not used analytical techniques that allowed the simultaneous analysis of related variables [19]. Our understanding of the individualized and unique reactions to the caregiving experience could be improved through a more methodologically rigorous assessment of how the predictors of caregiving relate directly and indirectly to each other and to caregiver outcomes [20].

Kinght and Sayegh [13] proposed a common core model framework in caregiving stress models, in which perceived burden mediates the effects of stressors on caregiving outcomes such as depression. This proposal is based on the following theoretical statements: 1) the consequences of stress are not only caused by the direct effect of the stressors [7], 2) perceived burden is the result of the appraisal of stressors [21], 3) the consequences of stress are the result of the appraisal of stressors [7] and 4) perceived burden is defined as directly proportional to depressive symptoms [3]. Therefore, perceived burden may buffer the effect of stressors on depressive symptoms. However, few authors have analysed this core model, and their findings have been heterogeneous, with studies showing mediation [9, 22], and no mediation [19]. Thus, more research is needed on this issue.

On the other hand, some authors have extended this previous model to include a mediation effect of perceived burden in the relation between cultural factors and caregiving outcomes. Thus, Kim et al. [23] proposed a theoretical model in which perceived burden mediates the effect of cultural factors and health outcomes. In the specific case of motives for caregiving and depression, Powers [24] documented that the relationship between motives for caregiving and depression is mediated by perceived burden. The role of perceived burden as a mediator is further supported by studies showing a relationship between motives for caregiving and perceived burden [8, 16, 25], as well as links between perceived burden and depression [12].

**Fig. 1:**
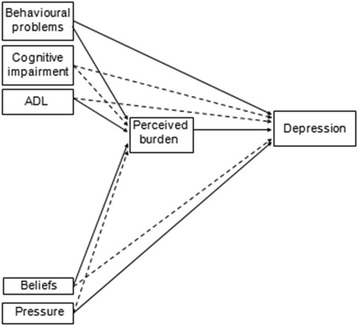
The starting model. *Notes.* ADL refers to the independence for the basic activities of daily living. Arrows with a dashed line show non-significant paths

In this paper, we aim to analyse the relationship between obligation to provide caregiving, distinguishing between beliefs of obligation and social pressure, and depressive symptoms in primary caregivers of disabled older relatives. We analyse these relationships while controlling for stressors because, as we said above, these variables are related to depression [3, 12]. Likewise, we take into account the simultaneous relationships among variables, because those related to informal care are often highly interrelated, as we said above. For this purpose, we propose the model of theoretical relationships shown in Fig. 1. This model is based on the conceptual models and findings mentioned above and has the following assumptions: perceived burden mediates the effects of stressors on depression, depression is negatively associated with beliefs of obligation and positively associated with social pressure, and perceived burden mediates the effects of beliefs of obligation and social pressure on depression.

Thus, in this study, the following hypotheses were tested:

H1: Depressive symptoms are associated with social pressure and with beliefs of obligation, while perceived burden mediating the effects of this factors on depressive symptoms.

This hypothesis is composed of three sub-hypotheses:

- H1.1: Depressive symptoms are negatively associated with beliefs of obligation.
- H1.2: Depressive symptoms are positively associated with social pressure.
- H1.3: Perceived burden mediates the effects of beliefs of obligation and social pressure on depressive symptoms.

H2: Perceived burden mediates the positive association between stressors and depressive symptoms.

## Methods

### Design, setting and sample

This study was conducted using a cross-sectional design. The study population consisted of the primary caregivers of disabled older relatives in the Health Care District of Jaén Norte (Andalusia, Spain). A sampling frame was created using the clinical records of older dependents who were cared for by a relative in the Primary Health Care Centres of the region (4223 subjects). We considered disabled older relatives to be older relatives who are dependent for at least one activity of daily living (basic or instrumental), and primary caregivers to be those taking the responsibility for care and delivering the largest amount of care.

We used a systematic random sampling with a sample size of 200 caregivers. Of the caregivers selected, none refused to participate in the study. The sample size (*N* = 200) allows the detection of a correlation coefficient of at least .17, with a confidence of 95% and a power of 80%. Regarding the path analysis, Nunnally and Bernstein [26] recommend a minimum of 10 participants per variable analysed. For this study, 28 participants per variable were included.

### Procedures

Data were collected through interviews. Data collection was carried out in 2013 (from January to February) by highly qualified nurses (nursing case managers with at least 3 years of experience or family nurses with at least 10 years of experience caring for the caregivers of disabled older relatives). These nurses had specific training in data collection (a 10-h training session) to ensure the quality and consistency of the data collection. The training session included recommendations about conducting interviews (context, introduction, and development), using the study’s measuring instruments (recommendations, demonstrations and queries), and coding of data (criteria and examples).

Initially, caregivers were contacted by their family nurses, who informed them about the study coinciding with a home visit, confirmed their participation, and dated the interview. Later, interviewers (other than the aforementioned family nurses) conducted the interviews in the caregivers’ homes.

We developed a pilot study (*n* = 20) for checking the data collection procedure and improving quality and uniformity of the data collection.

Confidentiality was guaranteed in the previous process and in the sampling process, and privacy was guaranteed during the interviews. Interviewers had no previous relation with the caregivers or the care recipients and did not know the study’s hypotheses.

### Measures

#### Beliefs of obligation and social pressure

The sense of obligation as a motive for caregiving was measured by two dimensions: an internal dimension represented by the *beliefs of obligation* and an external dimension represented by *social pressure*. Scales were developed in this study for measuring these issues. We developed both scales with three Likert scale items (Table 1) and four response options each item (4 = *strongly agree*, 1 = *strongly disagree*). Scores range from 3 to 12 in each dimension (a higher score reflects greater beliefs of obligation or social pressure). We developed these items from a literature review regarding the motives for caregiving and the results of 12 in-depth interviews with family caregivers of disabled elders. Two independent judges evaluated the literature and analysed the interviews through content analysis. At the end of the literature review and interviews, 16 items were generated, 11 for beliefs of obligation and 5 for social pressure. Then, we constructed a panel of 15 experts to analyse the adequacy of the items for the constructs measured. The panel of experts was constituted by health professionals with at least 5 years of experience in the care of caregivers of elderly people with disabilities. The experts were asked to express their agreement with the adequacy of the items for the construct measured. We selected items with an agreement of 70% or higher (6 items; 3 for beliefs of obligation and 3 for social pressure). The percentage of experts who considered each selected item as appropriate is shown in Table 1. For analysing the construct validity of the measure, we carried out an exploratory factor analysis with principal axis factors and direct oblimin rotation. In this analysis, two factors were obtained with eigenvalues higher than 1, which explained 61.7% of the variance. These factors agreed with the dimensions of beliefs of obligation and social pressure. Furthermore, a confirmatory factor analysis through Structural Equation Modelling (SEM) yielded excellent fit indices (X^2^ = 4.71, *p* = .58, df = 6, X2/df = 0.78, RMSEA = 0.000 (95% CI [0.000, 0.057], *p* = .92), SRMR = 0.019, CFI = 1.000). Regarding internal consistency, the Cronbach’s alpha values obtained were .74 for beliefs of obligation, .66 for social pressure, and .70 for both dimensions. Finally a pilot test was performed to assess the comprehension and acceptability of the scales by interviewing 20 family caregivers of disabled elderly.

**Table 1:** Items used to measure beliefs of obligation and social pressure and their results regarding content validity

Dimension	Items	% of adequacy
Beliefs of obligation	I care for him/her because I must follow the family tradition of caring for our relatives when they cannot care for themselves.	93%
	I care for him/her because, in my family, relatives have always been cared for when they could not care for themselves.	100%
	I care for him/her because I think we all have the obligation to care for our relatives when they cannot care for themselves.	100%
Social Pressure	I care for him/her because my family and people who I know expect it of me.	70%
	I care for him/her because I feel strongly forced by my family, neighbours and friends to take care of my relative.	80%
	I care for him/her because my family and the people who I know would not approve if I took my relative to a nursing home.	93%

Note. % of adequacy refers to the percentage of experts considering the item as adequate to the construct measured

#### Perceived burden

The perceived burden was measured by the Robinson’s Caregiver Strain Index (CSI) [27]. The CSI includes 13 items and measures perceived burden. Scores range from 0 to 13 (a higher score reflects a greater burden). It has been validated in the Spanish population with good results (high correlation with determinants and consequences of perceived burden; Cronbach’s alpha: .86) by López Alonso and Moral Serrano [28]. Cronbach’s alpha from our study was .76.

#### Stressors

Stressors are defined by the needs of the care recipient, which are divided into three: functional capacity, cognitive impairment and behavioral problems. These needs were measured by the Barthel Index (BI) [29], Pfeiffer’s Short Portable Mental Status Questionnaire (SPMSQ) [30], and Cummings’s Neuropsychiatric Inventory (NPI) [31].

The BI [29] is a 10-item scale with scores ranging from 0 to 100 that measures independence for the basic activities of daily living (ADL); the degree of independence is directly proportional to the test score. In this study, we used the version validated in Spain by Baztán et al. [32], which has good psychometric properties (high criterion validity, test-retest reliability of .98, and inter-observer reliability of .98). Cronbach’s alpha from our study was .91.

The SPMSQ [30] includes 10 items that measure cognitive impairment (range: 0–10; cognitive impairment is directly proportional to the test score). We used the version validated in Spain [33], which has a sensitivity of 85.7% and a specificity of 97.3%. Cronbach’s alpha from our study was .94.

The NPI [31] measures the frequency and severity of various behavioural problems represented by psychological and psychiatric symptoms (e.g., hallucinations, agitation, irritability). The scores range from 0 to 120 (a higher score reflects greater frequency and severity). We used the version validated in Spain by Vilalta-Franch et al. [34], which has an interobserver reliability of .93, a test-retest reliability for frequency of .79, and a test-retest reliability for severity of .86. Cronbach’s alpha from our study was .70.

#### Depression

Depressive symptoms was measured using the depression subscale of Goldberg’s Anxiety and Depression Scale [35]. This subscale consists of 9 items which are answered with “yes” or “no,” with 1 point given for each affirmative answer (range of 0 to 9 points; a higher score reflects greater depressive symptoms). The scale has been validated in the Spanish population [36] with good psychometric results (sensitivity, 83.1%; specificity, 81.8%; and positive predictive value, 95.3%). Cronbach’s alpha from our study was .90.

### Ethical considerations

This study was approved by the local Ethics Committee of the Research. Informed consent was obtained from the caregivers.

### Data analysis

Descriptive data analyses were performed for describing the sample and for assessing violations of the normality assumption. If the data were skewed, appropriate transformations were performed before the skewed variables were entered into the model. Bivariate analysis was carried out by calculating the Pearson’s linear correlation coefficient. In order to test the study hypotheses, path analysis was used. We chose path analysis because it allowed us to analyse our hypotheses while including mediation effects [37] and simultaneous estimation of the relationships among variables in order to estimate these relationships in an unbiased manner. To evaluate the model fit, the *p*-value, the normed chi-square (*X* ^*2*^/*df*), the Root Mean Square Error of Approximation (RMSEA) and its associated confidence interval, the standardized root mean square residual (SRMR), and the Comparative Fit Index (CFI) were used. We chose the previous fit indices because they have been found to be the least sensitive to sample size, model misspecification, and parameter estimates [38]. For a good fit of the model, the above measures should be as follows [37, 39]: *p* values above .10, *X* ^2^/*df* values below 2, RMSEA values below 0.08, SRMR values below 0.08, and CFI values above 0.95. For the various statistical tests, a significance level of .05 was used. The calculations were performed with Statistica 8 (descriptive analysis, transformation of variables, and bivariate analysis) and AMOS 18 (path analysis).

## Results

### Descriptive analysis

Descriptive data of the sample characteristics and the variables used in this study are showed in Table 2. The average age of the caregivers was 59.8 years (standard deviation = 12.9, range = 27 to 88). Most of the caregivers in the sample were women (81.0%) and offspring (62.0%). These features are similar to the last Spanish national sample analysed, in which there were 83.6% of women and 61.8% of offspring [40]. The average of depressive symptoms score for the caregivers was 2.94, based on a cut-off point of 3 (as Montón et al. recommended) [36] and 37% of the caregivers had depressive symptoms (95% CI [30.1, 43.9]). Regarding care-recipients, 60% of them had physical impairment and 40% had psychical impairment.

**Table 2:** Descriptive data of the sample characteristics and the variables used in this study

	*M*	*SD*	Theoretical range	Practical range	No.	%		95% CI
Caregiver age		59.8	12.9		27–88			
Caregiver gender	Female					162	81.0	[75.3, 86.7]
	Male					38	19.0	[13.3, 24.6]
Kinship	Spouse					59	29.5	[22.9, 36.1]
	Offspring					124	62.0	[55.0, 68.9]
	Other					17	8.5	[4.4, 12.6]
Depression		2.94	2.46	0–9	0–9			
Perceived burden		5.49	3.12	0–13	0–13			
Beliefs of obligation		9.20	3.08	3–12	3–12			
Social pressure		4.52	1.99	3–12	3–12			
Independence for the ADL		34.37	28.60	0–100	0–95			
Behavioural problems		14.18	14.88	0–120	0–100			
Cognitive impairment		5.09	3.49	0–10	0–10			

*Note*. ADL refers to the basic activities of daily living

Regarding bivariate correlation analysis (Table 3), depressive symptoms was positively correlated with perceived burden, social pressure, and behavioural problems.

**Table 3:** Correlation matrix of the variables used in this study

	2	3	4	5	6	7
1 Depression	.44**	−.08	.18*	−.04	.31**	.044
2 Perceived burden		−.23**	−.09	−.29**	.32**	.14*
3 Beliefs of obligation			.34**	.01	−.07	−.05
4 Social pressure				.13	.04	−.07
5 Independence for the ADL					−.15*	−.55**
6 Behavioural problems						.14*
7 Cognitive impairment						

*Note.* ADL refers to the basic activities of daily living

**p < .01. *p < .05

### Path analysis

**Fig. 2:**
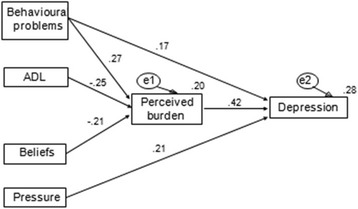
The final model. *Notes.* ADL refers to the independence for the basic activities of daily living. The numbers by the arrows represent standardized regression coefficients. The numbers at the upper right corners of the boxes represent r^2^ of the partial regression of each variable; e1 and e2 are latent errors. Only paths significant at *p* < 0.05 were taken into account

The hypothesized model contained several non-significant paths (see dashed lines in Fig. 1). For parsimony, the paths that were not statistically significant were removed from this model, resulting in a second, final model (Fig. 2) with excellent fit indices (*X* ^*2*^ = 2.30, *p* = .51, *df* = 3, X^2^/*df* = 0.77, RMSEA = 0.000 (95% CI [0.000, 0.10], *p* = .69), SRMR = 0.019, CFI = 1.000). Table 4 shows the direct, indirect, and total effects on depression. The final model explained 28% of the variance of depression. When an alternative model without mediation effects of burden was tested, poor fit indices were found (*X* ^*2*^ = 35.269, *p* < .001, *df* = 4, *X* ^*2*^/*df* = 8.81, RMSEA = 0.20, SRMR = 0.10, CFI = 0.75).

**Table 4:** Standardized effects of the variables on depression in the final model (Fig. 2)

	Direct effects	Indirect effects	Total effects
Perceived burden	.42**	.00	.42*
Beliefs of obligation	.00	−.21*	−.21*
Social pressure	.21**	.00	.21*
Behavioural problems	.17**	.11*	.28*
Independence for the ADL	.00	−.11*	−.11*

*Notes*. The level of significance of indirect effects and total effects is calculated by bootstrapping. ADL refers to the basic activities of daily living

**p < .01. *p < .05

Hypothesis H1 predicted that depressive symptoms would be negatively associated with beliefs of obligation (H1.1) and positively associated with social pressure (H1.2), with perceived burden mediating the effects of beliefs of obligation and social pressure on depressive symptoms (H1.3). This hypothesis was partially supported since depressive symptoms were positively associated with social pressure (β = .21) and negatively associated with beliefs of obligation via perceived burden (indirect effect = −.21).

Hypothesis H2 indicated that perceived burden would mediate the positive association between stressors and depressive symptoms. This hypothesis was partially supported. Depressive symptoms was positively associated with behavioural problems both directly (β = .17) and indirectly via perceived burden (indirect effects = .11) and was negatively associated with independence for the ADL via perceived burden (indirect effects = −.11).

## Discussion

In this study, depressive symptoms had a direct and positive association with perceived burden, behavioural problems and social pressure. It was indirectly related through perceived burden to behavioural problems, independence for the ADL and beliefs of obligation.

### Hypothesis H1

Our results partially supported the hypothesis H1, which stated that depressive symptoms would be negatively associated with beliefs of obligation and positively associated with social pressure, with perceived burden mediating the effects of beliefs of obligation and social pressure on depressive symptoms. Social pressure as a motive for caregiving was related to greater depressive symptoms (sub-hypothesis H1.1 supported), while beliefs of obligation were related to reduce depressive symptoms (sub-hypothesis H1.2 supported), with their effects mediated by perceived burden (sub-hypothesis H1.3 partially supported).

Our findings showed the importance of cultural factors in the caregiving process that have been argued by several authors [41, 13] and supported previous evidence that these cultural factors influence the perception of caregiving situations [42, 8] and the health outcomes of caregiving [15].

The final model supports the idea that social pressure does not influence perceived burden because social pressure may not affect the appraisal of the care situation. Instead, social pressure may act directly on the state of mind, as documented in two previous studies. First, a study by Mirowsky and Ross [43] suggested that depression is the result of socio-structural pressures, and second, a study by Ramos-Diaz [44] that suggests that social pressure is an additional source of stress that increases emotional arousal, generating feelings of discomfort.

Our results agree with the results of Romero-Moreno et al. [15] and support the multidimensionality of the concept of obligation argued in previous works [8, 16]. Theoretically, the multidimensionality of obligation is consistent with the Ryan and Deci’s self-determination theory [17].

Our findings also suggest that when the obligation for caregiving has an internal nature, it might offer protection from the negative consequences of caregiving; but when it has an external nature, it might increase the risk of such consequences. Our results also help to explain the heterogeneity of results in previous analyses of obligation and negative consequences of caregiving [16]. In this sense, it is possible that the different dimensions of obligation can vary from one study to another due to the different relationships between each dimension and the negative effects of caregiving. Our study and the study by Romero-Moreno et al. [15] have cross-sectional designs, therefore, causal conclusions cannot be revealed clearly. Thus, more research is needed on the relationship between social pressure and depressive symptoms.

Given that internal and external obligations are related differently with depressive symptoms, each dimension should be considered separately when developing interventions for preventing depressive symptoms. Thus, in order to use the concept of obligation for preventing depression, it may be useful to discriminate between internal and external obligation (beliefs of obligation and social pressure, respectively). Discriminating internal and external obligation may help both clinician and researchers to identify a profile of caregivers at risk of depression. This discrimination is supported by findings of other authors who found greater distress [15], greater burden [45] and lower caregiving satisfaction [46] in those with external obligation compared to those having internal obligation.

### Hypothesis H2

Our results partially supported the hypothesis H2 (the mediation of the effects of stressors on depressive symptoms by perceived burden). Behavioural problems had both direct and indirect effects (mediated by perceived burden) on depressive symptoms, and independence for the ADL had only an indirect effect on depressive symptoms, also mediated by perceived burden. These results agreed with the results of other studies that analysed effects regarding both psychological health [47] and depression [9, 22, 45, 48]. Our findings can be explained by the fact that perceived burden is the result of the appraisal of the caregiving stressors [21]. Thus, to appraise the situation as harmful and overwhelming may lead to more perceived burden, and this perceived burden may increase the risk of depression.

This study has the following strengths: (a) the analysis of a probability sample avoids the appearance of selection biases associated with convenience samples, which are quite common in the caregiving literature [49]; (b) the use of a sufficient sample size prevents the onset of Type II errors; (c) the analysis used allows the study of simultaneous interrelationships among variables and prevents confounding effects.

This study has two limitations. First, the limitation of being cross-sectional, which does not allow it to establish causal relationships. Second, the lack of caregivers in the expert panel.

## Conclusions

Despite the above limitations, we can establish the following conclusions: (a) our results support the multidimensional concept of obligation, suggesting the existence of both an external obligation (social pressure) and an internal obligation (beliefs of obligation); (b) our findings support the hypothesis that external obligation is related to negative caregiving consequences, while internal obligation protects from these consequences; specifically, in the sample studied, social pressure is positively related to depressive symptoms, while beliefs of obligation are negatively related to perceived burden; (c) our findings support the partial mediation of stressors on depression by perceived burden.

The above findings support the importance of understanding the obligation for caregiving in the caregiving process. Particularly, it may be useful to discriminate between internal (beliefs of obligation) and external (social pressure) obligation for care during the assessment of primary caregivers of disabled older relatives for a better understanding of the caregiving process, as well as to support prevention and early detection of negative caregiving consequences such as depression.

Future research regarding obligation for caregiving must consider that obligation is a multidimensional concept with two dimensions (internal and external) that may differentially affect depression.

## Abbreviations

ADL: Basic activities of daily living
BI: Barthel Index
CFI: Comparative Fit Index
CSI: Caregiver Strain Index
NPI: Cummings’s Neuropsychiatric Inventory
RMSEA: Root Mean Square Error of Approximation
SEM: Structural Equation Modelling
SPMSQ: Pfeiffer’s Short Portable Mental Status Questionnaire
SRMR: Standardized root mean square residual
